# Improved Benefit Risk Profile of Rivaroxaban in a Subpopulation of the MAGELLAN Study

**DOI:** 10.1177/1076029619886022

**Published:** 2019-11-20

**Authors:** Alex C. Spyropoulos, Concetta Lipardi, Jianfeng Xu, Wentao Lu, Eunyoung Suh, Zhong Yuan, Bennett Levitan, Chiara Sugarmann, Yoriko De Sanctis, Theodore E. Spiro, Elliot S. Barnathan, Gary E. Raskob

**Affiliations:** 1 The Donald and Barbara Zucker School of Medicine at Hofstra/Northwell, Hempstead, NY, USA; 2 The Feinstein Institute for Medical Research, Manhasset, NY, USA; 3 Department of Medicine, Anticoagulation and Clinical Thrombosis Services Northwell Health at Lenox Hill Hospital, NY, USA; 4 Janssen Research & Development, LLC, Raritan, NJ, USA; 5 Clinical Development, Pharmaceuticals, Bayer U.S. LLC, Whippany, NJ, USA; 6 College of Public Health, The University of Oklahoma Health Sciences Center, Oklahoma City, OK, USA

**Keywords:** direct oral anticoagulants, medical illness, venous thromboembolism, risk factors, hemorrhage, thrombosis prophylaxis

## Abstract

Acutely ill medical patients are at risk of venous thromboembolism (VTE) and VTE-related mortality during hospitalization and posthospital discharge, but widespread adoption of extended thromboprophylaxis has not occurred. We analyzed a subpopulation within the MAGELLAN study of extended thromboprophylaxis with rivaroxaban to reevaluate the benefit risk profile. We identified 5 risk factors for major and fatal bleeding after a clinical analysis of the MAGELLAN study and analyzed efficacy and safety with these patients excluded (n = 1551). Risk factors included: active cancer, dual antiplatelet therapy at baseline, bronchiectasis/pulmonary cavitation, gastroduodenal ulcer, or bleeding within 3 months before randomization. We evaluated efficacy, safety, and benefit risk using clinically comparable endpoints in the subpopulation. At day 10, rivaroxaban was noninferior to enoxaparin (relative risk [RR] = 0.82, 95% confidence interval [CI] = 0.58-1.15) and at day 35 rivaroxaban was significantly better than enoxaparin/placebo (RR = 0.68, 95% CI = 0.53-0.88) in reducing VTE and VTE-related death. Major bleeding was reduced at day 10 (RR = 2.18, 95% CI = 1.07-4.44 vs 1.19, 95% CI = 0.54-2.65) and at day 35 (2.87, 95% CI = 1.60-5.15 vs 1.48, 95% CI = 0.77-2.84) for MAGELLAN versus this subpopulation, respectively. The benefit risk profile was favorable in this subpopulation treated for 35 days, with the number needed to treat ranging from 55 to 481 and number needed to harm from 455 to 1067 for all pairwise evaluations. Five exclusionary criteria defined a subpopulation of acutely ill medical patients with a positive benefit risk profile for in-hospital and extended thromboprophylaxis with rivaroxaban.

## Introduction

Venous thromboembolism (VTE) in hospitalized medical patients represents a leading cause of premature death and major morbidity worldwide.^1^ It is estimated that approximately 75% of autopsy-proven fatal pulmonary embolic (PE) events in hospitalized patients occur in nonsurgical acutely ill medical populations^2^ and these patients tend to develop more severe forms of VTE (including nonfatal and fatal PE) compared to their surgical counterparts.^3,4^ A significant proportion of the more than 20 million patients hospitalized for an acute medical illness in the United States of America (US) and the European Union (EU) remain at risk of VTE.^5,6^

Although evidence-based antithrombotic guidelines recommend that thromboprophylaxis be given to hospitalized medical patients at increased risk of VTE until the patient is fully mobile or discharged from the hospital,^7^ recent longitudinal studies suggest that the period of VTE risk extends far beyond the period of hospitalization and that approximately 80% of VTE events now occur within 6 weeks of hospital discharge.^8,9^ Previous trials of extended thromboprophylaxis (ie, beyond hospital discharge) with low-molecular-weight heparin or direct oral anticoagulants (DOACs) have demonstrated prevention of VTE events.^10–13^ However, this was associated with an increase in the risk of bleeding events. Largely because of this excess bleeding and suboptimal benefit risk profile, antithrombotic guidelines have suggested against routine use of extended thromboprophylaxis in medically ill patients.^7,14,15^ However, more recent large trials of DOACs in this setting have shown that with careful selection criteria, a population of medically ill patients with a low bleeding risk can be identified that would likely benefit from a strategy of extended thromboprophylaxis.^16,17^

Optimizing the benefit risk profile of extended thromboprophylaxis in acutely ill medical patients thus remains an important unmet clinical need, both by improved patient selection to define an at-risk group that would benefit from extended thromboprophylaxis as well as by excluding patients at high risk of bleeding.^18^ The MAGELLAN trial was a randomized study to compare the efficacy and safety of extended thromboprophylaxis in hospitalized medical patients with rivaroxaban 10 mg once daily for 35 ± 4 days versus standard of care with enoxaparin 10 ± 4 days followed by placebo.^12^ Rivaroxaban was noninferior to enoxaparin and superior to placebo for reducing the risk of VTE but was associated with more than a 2-fold increased risk of bleeding, including fatal bleeding.^12^ In 2012-2013, when the results became known, an investigation of risk factors associated with major bleeding was undertaken. The knowledge gained was incorporated as additional exclusion criteria into the follow-on trial, called MARINER, to improve the safety profile of rivaroxaban for extended thromboprophylaxis in this population. Before the results of the MARINER study were available, we conducted a post hoc retrospective analysis to assess whether excluding patients with these key risk factors for major bleeding would be able to define a subpopulation of the MAGELLAN trial with an improved benefit risk profile in acutely ill medical patients.

## Materials and Methods

### Study Design

The MAGELLAN protocol and results have been reported previously.^19,12^ Briefly, the MAGELLAN study (NCT00571649) was a multicenter, randomized, double-blind, parallel-group efficacy and safety study comparing rivaroxaban (10 mg once daily) administered for 35 ± 4 days to enoxaparin (40 mg once daily) administered for 10 ± 4 days followed by placebo, for the prevention of VTE in hospitalized acutely ill medical patients during the in-hospital and posthospital discharge period. Eligible patients included adults who were at least 40 years of age, hospitalized for an acute medical illness (ie, heart failure, active cancer, acute ischemic stroke, acute infectious and inflammatory disease, and acute respiratory insufficiency), at risk of VTE due to moderate or severe immobility, and had additional risk factors for VTE such as prolonged immobilization, age ≥75 years, history of cancer, history of VTE, history of heart failure, thrombophilia, acute infectious disease contributing to the hospitalization, and body mass index ≥35 kg/m^2^.

The MAGELLAN subpopulation, which was not prespecified as a part of the original trial methodology, consisted of patients in the MAGELLAN trial without any of the following 5 risk factors for major bleeding: active cancer at randomization, dual antiplatelet therapy at baseline, a medical history of bronchiectasis/pulmonary cavitation, active gastroduodenal ulcer or any bleeding in the previous 3 months prior to randomization. Patients who met one or more of these criteria prior to or at randomization using standardized MedDRA queries or preferred terms of MedDRA version 13.1 based on data entered into the case report forms in the original MAGELLAN database were excluded from this MAGELLAN subpopulation. These 5 exclusion criteria were part of the MARINER study (NCT02111564) that tested extended thromboprophylaxis with rivaroxaban in the posthospital discharge period. The MARINER protocol was reported previously,^20^ and the results have been recently published.^17^

### Study Outcomes

The efficacy and safety outcomes assessed in the MAGELLAN subpopulation are the same as specified in the MAGELLAN trial. Briefly, the coprimary efficacy outcomes were the composite of asymptomatic proximal deep vein thrombosis (DVT) in lower extremity detected by mandatory bilateral lower extremity venous ultrasonography, symptomatic DVT in the lower extremity, proximal or distal, symptomatic, nonfatal PE, and VTE-related death at day 10 and day 35. The primary population for the evaluation of superiority testing was the modified intent to treat (mITT) population, while the primary population used for noninferiority testing was the per protocol population. The mITT population included patients who were valid for the safety analysis with an adequate ultrasonography assessment of VTE at day 35. The per protocol population included who were valid for the mITT analysis and had an adequate assessment of VTE that was done not later than 48 hours after the stop of study drug and met the inclusion criteria regarding medical illness and VTE risk factors and had no major protocol deviation as defined in the statistical analysis plan. The events were assessed by the Ultrasonography Adjudication Committee and the Clinical Events Adjudication Committee. The principal safety outcomes were the incidence of treatment-emergent clinically relevant bleeding defined as the composite of treatment-emergent major bleeding using International Society of Thrombosis and Haemostasis definitions and nonmajor clinically relevant (NMCR) bleeding at day 10 and day 35.^20^ These endpoints were assessed from the first dose of study drug until the end of treatment plus 2 days.

The benefit risk profile was conducted using 5 pairwise comparisons of composite efficacy and safety outcomes, a structure that takes into consideration similar degrees of clinical severity and impact.^7,21,22^ The first pair (pair 1), conducted in the mITT population, included asymptomatic proximal DVT, symptomatic, and fatal VTE versus major bleeding events. The rest of the pairs were conducted in the safety population through day 35 for both efficacy and safety, regardless if treatment was stopped prematurely. The second pair (pair 2) included symptomatic and fatal VTE versus major bleeding, while the other 3 pairs mainly focused on events in the primary composite efficacy and principal safety endpoints that were fatal or caused irreversible harm: nonfatal PE and VTE-related death versus critical site bleeding and fatal bleeding (pair 3), nonfatal PE, myocardial infarction (MI), nonhemorrhagic stroke, cardiovascular (CV), and VTE-related death versus critical site bleeding and fatal bleeding (pair 4) and VTE-related death versus fatal bleeding (pair 5). The data scopes analyzed were selected to compare short-term thromboprophylaxis with enoxaparin (at day 10) and extended-duration thromboprophylaxis with rivaroxaban (at day 35).

### Statistical Analyses

Selected statistical analyses to evaluate efficacy and safety presented in this MAGELLAN subpopulation were rerun using the original study definitions, data rules, and derivations as outlined in the MAGELLAN Statistical Analysis Plan. Briefly, in all the analysis results presented here, relative risks (RR), including their corresponding confidence intervals, were calculated using the Mantel-Haenszel method available in PROC FREQ of SAS version 9.4.^23^ If not specified otherwise, the nominal 95% confidence intervals were presented.

The Firth’s logistic regression model was used to test the homogeneity of treatment effect in treatment-emergent major bleeding for the MAGELLAN subpopulation and non-MAGELLAN subpopulation.^24^ The *P* value for the coefficient of treatment-subpopulation interaction in the model is .02, indicating that the treatment effect in the MAGELLAN subpopulation is different from the treatment effect in the non-MAGELLAN subpopulation. This result reinforces the intention of reducing major bleeding risk in the rivaroxaban group over the enoxaparin group within this MAGELLAN subpopulation.

To simplify interpretation of multiple treatment difference simultaneously and to avoid the differences in the background event rate across different types of outcome events that can make RR ratios difficult to compare, the risk difference (ie, difference in incident rates) or excess number of events has been used to evaluate the benefit risk profile. Excess number of events was defined as the risk difference times a hypothetical population size (ie, 10 000 patients). In addition, the number-needed-to-treat to benefit (NNT) or harm (NNH) were used to quantify benefits and risks as additional metrics, respectively, which were calculated as the reciprocal of the risk differences.

## Results

### Baseline Patient Characteristics

The MAGELLAN study randomized 8101 patients from 562 sites in 52 countries and the safety population of patients who received at least one dose of study medication was 7998 patients.^12^ The 5 key risk factors for major bleeding events in MAGELLAN were identified using study data forms as described in section “Materials and Methods.” With the retrospective use of these risk factors, 1551 patients were excluded from MAGELLAN and the remaining 6447 patients, approximately 80% of MAGELLAN, represented the MAGELLAN subpopulation (Supplementary Table 1). Of the excluded patients, 584 (38%) had active cancer, 479 (31%) took dual antiplatelet therapy at baseline, 121 (8%) had baseline bronchiectasis, 232 (15%) had a history within 3 months of randomization of gastrointestinal ulcer, and 259 (17%) had bleeding within 3 months prior to randomization (Supplementary Table 2).

No differences for disposition, demographics, and other baseline characteristics were observed in this subpopulation when compared with the original population. In general, patients in both treatment groups had a similar medical history and baseline pretreatment hospitalization characteristics. Over 99% of patients had complete immobilization or decreased mobility, and the median length of hospital stay was 11 days. Reasons for hospitalization and additional risk factors for thromboembolism were similar between the treatment groups. The causes of hospitalization were acute infectious diseases (48.1%) followed by heart failure (34.6%), acute respiratory insufficiency (29.0%), stroke (18.1%), and acute inflammatory and rheumatic diseases (4.0%) and were balanced between treatment groups (Table 1). There were no patients with active cancer as admitting diagnosis because this was one of the criteria used to exclude patients from the MAGELLAN subpopulation. All the patients had at least one additional VTE risk factor. The most represented factors were age ≥75 years (∼40%) and a history of heart failure (∼37%). No appreciable differences between treatment groups were noted for prior and concomitant medications. In all phases of the study, median duration of treatment and compliance was similar between treatment groups (Table 1). The duration of follow-up was similar between treatment groups for all analysis populations. Using the modified IMPROVE risk score, slightly more than half of patients were at moderate risk (ie, IMPROVE 2 or 3), while approximately one-third were at high risk (≥4).

**Table 1.** Demographics and Baseline Characteristics (Safety Analysis Set).

	MAGELLAN		MAGELLAN Subpopulation	
Treatment Group	Rivaroxaban, N = 3997, n (%)	Enoxaparin, N = 4001, n (%)	Rivaroxaban, N = 3218, n (%)	Enoxaparin, N = 3229, n (%)
Sex (male)	2223 (55.6)	2103 (52.6)	1741 (54.1)	1661 (51.4)
Race				
White	2749 (68.8)	2744 (68.6)	2262 (70.3)	2245 (69.5)
Black	89 (2.2)	92 (2.3)	72 (2.2)	75 (2.3)
Asian	793 (19.8)	794 (19.8)	588 (18.3)	618 (19.1)
Native American	12 (0.3)	12 (0.3)	10 (0.3)	12 (0.4)
Hispanic	69 (1.7)	70 (1.7)	66 (2.1)	65 (2.0)
Uncodable	106 (2.7)	112 (2.8)	86 (2.7)	87 (2.7)
Age (years) mean ± SD	69.2 ± 11.9	69.2 ± 11.7	69.6 ± 11.9	69.6 ± 11.6
Age groups, n				
< 65 years	1323 (33.1)	1363 (34.1)	1035 (32.2)	1064 (33.0)
65 to < 75 years	1144 (28.6)	1090 (27.2)	902 (28.0)	871 (27.0)
≥ 75 years	1530 (38.3)	1548 (38.7)	1281 (39.8)	1294 (40.1)
Body mass index (kg/m^2^), mean ± SD	28.2 ± 7.3	28.2 ± 7.2	28.7 ± 7.4	28.6± 7.4
Duration of treatment (days), mean ± SD	29.0 ± 11.6	8.3 ± 2.8	29.3 ± 11.3	8.3± 2.8
Duration of follow-up (days), mean ± SD	59.2 ± 10.4	58.9 ± 10.0	59.2 ± 10.2	58.9 ± 9.9
Compliance percentage, mean ± SD	98.5 ± 7.4	99.3 ±4.2	98.6 ± 7.4	99.4 ± 4.2
Creatinine clearance				
< 30 mL/min	81 (2.0)	64 (1.6)	67 (2.1)	51 (1.6)
30 to < 50 mL/min	780 (19.5)	804 (20.1)	637 (19.8)	662 (20.5)
50 to 80 mL/min	1487 (37.2)	1536 (38.4)	1191 (37.0)	1234 (38.2)
> 80 mL/min	1571 (39.3)	1522 (38.0)	1265 (39.3)	1228 (38.0)
Reason of hospitalization				
Heart failure (NYHA class III or IV)^a^	1292 (32.3)	1301 (32.5)	1104 (34.3)	1129 (35.0)
Active cancer^b^	294 (7.4)	290 (7.2)	0	0
Acute ischemic stroke^b^	691 (17.3)	692 (17.3)	585 (18.2)	585 (18.1)
Acute infectious and inflammatory diseases	1904 (47.6)	1876 (46.9)	1633 (50.7)	1597 (49.5)
Acute infectious disease	1826 (45.7)	1801 (45.0)	1566 (48.7)	1535 (47.5)
Acute inflammatory or rheumatic disease	150 (3.8)	149 (3.7)	131 (4.1)	127 (3.9)
Acute respiratory insufficiency	1085 (27.1)	1151 (28.8)	918 (28.5)	950 (29.4)
Risk factor for VTE				
Severe varicosis	494 (12.4)	459 (11.5)	436 (13.5)	396 (12.3)
Chronic venous insufficiency	612 (15.3)	571 (14.3)	537 (16.7)	486 (15.1)
History of cancer	690 (17.3)	666 (16.6)	352 (10.9)	328 (10.2)
History of DVT or PE	196 (4.9)	178 (4.4)	165 (5.1)	151 (4.7)
Heart failure history (NYHA III/IV)	1391 (34.8)	1370 (34.2)	1193 (37.1)	1190 (36.9)
Thrombophilia	15 (0.4)	9 (0.2)	13 (0.4)	7 (0.2)
Recent major surgery (6 to 12 weeks)	29 (0.7)	32 (0.8)	17 (0.5)	17 (0.5)
Recent serious trauma (6 to 12 weeks)	7 (0.2)	7 (0.2)	3 (<0.1)	6 (0.2)
Hormone replacement therapy	48 (1.2)	46 (1.1)	36 (1.1)	38 (1.2)
Age ≥ 75 years	1530 (38.3)	1548 (38.7)	1281 (39.8)	1294 (40.1)
Morbid obesity (BMI ≥ 35 kg/m^2^)	604 (15.1)	612 (15.3)	534 (16.6)	544 (16.8)
Acute infectious disease contributing to hospitalization	558 (14.0)	594 (14.8)	451 (14.0)	483 (15.0)
Modified IMPROVE Score^c^				
1 (low risk)	501 (12.5)	501 (12.5)	445 (13.8)	452 (14.0)
2,3 (moderate risk)	2114 (52.9)	2164 (54.1)	1765 (54.8)	1796 (55.6)
≥4 (high risk)	1382 (34.6)	1336 (33.4)	1008 (31.8)	981 (30.4)

Abbreviations: DVT, deep vein thrombosis; NYHA, New York Heart Association; PE, pulmonary embolism; SD, standard deviation, VTE, venous thromboembolism.

^a^ Patients with heart failure (NYHA class III or IV), previous hospitalizations for heart failure (NYHA class III or IV), or were chronically in NYHA class III or IV status.

^b^ Patients with active cancer, and patients with acute ischemic stroke with lower extremity paresis or paralysis were not required to have an additional risk factor.

^c^ Modified IMPROVE Score was calculated as noted for the MARINER study.^20^

### Efficacy Outcomes

In the MAGELLAN subpopulation, the incidence of the primary efficacy endpoint outcome at day 10 (per protocol analysis set) was 2.4% (58/2385) in the rivaroxaban group and 3.0% (72/2433) in the enoxaparin group (RR = 0.82, 95% confidence intervals [CI] = 0.58-1.15). Given that the noninferiority margin was 1.5 in the original MAGELLAN study, it is reasonable to conclude that rivaroxaban is likely to be noninferior to enoxaparin in this subpopulation (Table 2). The incidence of the primary efficacy outcome at day 35 (mITT analysis set) was significantly lower in the rivaroxaban group (3.9% [94/2419]) than in the enoxaparin group (5.7% [143/2506], RR = 0.68, 95% CI = 0.53-0.88, *P* = .0029). The predominant event in both treatment groups was asymptomatic lower extremity proximal DVT (rivaroxaban, 3.0%; enoxaparin, 4.4%). The rivaroxaban group had a numerically lower incidence of VTE-related death (0.6%) than the enoxaparin group (1.0%).

**Table 2.** Key Efficacy Results in the MAGELLAN and in the MAGELLAN Subpopulation.

Endpoint/Components	MAGELLAN			MAGELLAN Subpopulation		
Modified ITT, day 35	Rivaroxaban N = 2967, n (%)	Enoxaparin N = 3057, n (%)	RR (95% CI)	Rivaroxaban N = 2419, n (%)	Enoxaparin N = 2506, n (%)	RR (95% CI)
Any event	131 (4.4)	175 (5.7)	0.77 (0.62-0.96)	94 (3.9)	143 (5.7)	0.68 (0.53-0.88)
Symptomatic lower extremity DVT	13 (0.4)	15 (0.5)		9 (0.4)	10 (0.4)	
Symptomatic nonfatal PE	10 (0.3)	14 (0.5)		7 (0.3)	10 (0.4)	
Asymptomatic proximal DVT	103 (3.5)	133 (4.4)		73 (3.0)	110 (4.4)	
VTE-related death	19 (0.6)	30 (1.0)		15 (0.6)	26 (1.0)	
Per protocol, day 10	Rivaroxaban, N = 2938, n (%)	Enoxaparin, N = 2993, n (%)	RR (95% CI)	Rivaroxaban N = 2385, n (%)	Enoxaparin N = 2433, n (%)	RR (95% CI)
Any event	78 (2.7)	82 (2.7)	0.97 (0.71-1.31)	58 (2.4)	72 (3.0)	0.82 (0.58-1.15)
Symptomatic lower extremity DVT	7 (0.2)	6 (0.2)		6 (0.3)	4 (0.2)	
Symptomatic nonfatal PE	6 (0.2)	2 (<0.1)		5 (0.2)	2 (<0.1)	
Asymptomatic proximal DVT	71 (2.4)	71 (2.4)		52 (2.2)	62 (2.5)	
VTE-related death	3 (0.1)	6 (0.2)		2 (<0.1)	6 (0.2)	

Abbreviations: CI, confidence interval; DVT, deep vein thrombosis; mITT, modified intent to treat; PE, pulmonary embolism; RR, relative risk; VTE, venous thromboembolism.

### Safety Outcomes

In the MAGELLAN subpopulation, the safety of rivaroxaban compared with enoxaparin through day 35 was improved. In MAGELLAN, the incidence of major bleeding was 1.1% (43/3997) in the rivaroxaban group and 0.4% (15/4001) in the enoxaparin-placebo group (RR = 2.87, 95% CI = 1.60-5.15). In the MAGELLAN subpopulation, the incidence of major bleeding was 0.7% (22/3218) in the rivaroxaban group and 0.5% (15/3229) in the enoxaparin/placebo group (RR = 1.48, 95% CI = 0.77-2.84). The RR for major bleeding in the rivaroxaban group was also reduced from 2.18 (95% CI = 1.07-4.44) to 1.19 (95% CI = 0.54-2.65) at day 10 in the entire population versus the subpopulation, respectively. At day 35, the number of fatal bleeds with rivaroxaban were reduced from 7 cases to 3 in MAGELLAN and the MAGELLAN subpopulation (0.2% vs <0.1%, respectively), while there was one fatal bleed in the enoxaparin/placebo group. At day 10, the number of fatal bleeds with rivaroxaban were reduced from 5 cases to 1 in MAGELLAN and MAGELLAN subpopulation, respectively (0.1% vs <0.1%), while there was 1 fatal bleed in the enoxaparin/placebo group for the entire population and the subpopulation. In both MAGELLAN and the MAGELLAN subpopulation, there were 2 versus 0 fatal bleeds in the postdischarge phase for the rivaroxaban versus enoxaparin/placebo groups, respectively (Table 3 and Supplementary Table 3).

**Table 3.** Key Safety Results in the MAGELLAN Study and the MAGELLAN Subpopulation (Safety Analysis Set).

Bleeding Event by Treatment Phase	MAGELLAN			MAGELLAN Subpopulation		
	Rivaroxaban, N = 3997, n (%)	Enoxaparin, N = 4001, n (%)	RR (95% CI)	Rivaroxaban, N = 3218, n (%)	Enoxaparin, N = 3229, n (%)	RR (95% CI)
Rivaroxaban-enoxaparin/placebo treatment phase (day 1 to day 35)						
Clinically relevant bleeding	164 (4.1)	67 (1.7)	2.46 (1.85-3.25)	114 (3.5)	49 (1.5)	2.35 (1.69-3.26)
Major bleeding	43 (1.1)	15 (0.4)	2.87 (1.60-5.15)	22 (0.7)	15 (0.5)	1.48 (0.77-2.84)
Clinically relevant nonmajor bleeding	124 (3.1)	52 (1.3)		93 (2.9)	34 (1.1)	
Fatal bleeding	7 (0.2)	1 (<0.1)		3 (<0.1)	1 (<0.1)	
Rivaroxaban-enoxaparin treatment phase (day 1 to day 10)						
Clinically relevant bleeding	111 (2.8)	49 (1.2)	2.27 (1.63-3.17)	80 (2.5)	35 (1.1)	2.31 (1.56-3.42)
Major bleeding	24 (0.6)	11 (0.3)	2.18 (1.07-4.44)	13 (0.4)	11 (0.3)	1.19 (0.54-2.65)
Clinically relevant nonmajor bleeding	88 (2.2)	38 (0.9)		67 (2.1)	24 (0.7)	
Fatal bleeding	5 (0.1)	1 (<0.1)		1 (<0.1)	1 (<0.1)	

Abbreviations: CI, confidence interval; RR, relative risk.

No significant reduction of the number of NMCR bleeding was observed in the MAGELLAN subpopulation when compared to MAGELLAN at day 35 (3.1% vs 1.3% in MAGELLAN; 2.9% vs 1.1% in the subpopulation, for rivaroxaban and enoxaparin/placebo groups, respectively). For clinically relevant bleeding (the composite of major and NMCR bleeding) at day 35, the RR was similar in the MAGELLAN population (4.1% vs 1.7%, RR = 2.46, 95% CI = 1.85-3.25) and the subpopulation (3.5% vs 1.5%, RR = 2.35, 95% CI = 1.69-3.26) for the rivaroxaban and enoxaparin/placebo groups, respectively (Table 3). Treatment-emergent clinically relevant bleeding events remained higher in the rivaroxaban group compared to the enoxaparin/placebo group mainly due to NMCR.

### Benefit Risk Profile

For the benefit risk analysis, we utilized the same time period and prevented double counting for fatal bleeds. Hence all bleeds through day 35 were included (even if off treatment), and bleeding deaths were not counted as CV deaths. As the primary analysis was in the mITT population, the first pair compared efficacy and safety in this population. Using the primary endpoint, there would be 131 fewer event in the rivaroxaban group with an NNT of 77 to prevent one event, but this was counterbalanced by causing 51 more major bleeds (Table 4). The benefit risk profile was improved in the subpopulation, with 182 events prevented and only 18 major bleeds caused, with NNT and NNH of 55 and 560, respectively. A similar pattern can be observed for the other benefit risk pairs analyzed in the safety population as only symptomatic events were considered. In all 5 pairs, more ischemic events were prevented than hemorrhagic events caused in the subpopulation (Table 4). For 35 days of treatment, the number need to treat to prevent 1 event ranged from 55 to 481 for the 5 pairs, while the number needed to harm ranged from 455 to 1067.

**Table 4.** Benefit Risk MAGELLAN and MAGELLAN Subpopulation at Day 35.

Pair	Outcome	Rivaroxaban	Enoxaparin	Rivaroxaban-Enoxaparin	NNT or NNH^a^
		# of Patients With Event (Crude Rate in %)	# of Patients With Event (Crude Rate in %)	Excess Number of Events for 10 000 Patients [95% CI]	
MAGELLAN					
		(N = 2967) mITT	(N = 3057) mITT		
1	Asymptomatic lower proximal DVT, symptomatic VTE (nonfatal), and VTE-related death	131 (4.42)	175 (5.72)	−131 [−242 to −20]	−77
	Major bleeding	24 (0.81)	9 (0.29)	51 [14to 89]	194
		(N = 3997) Safety	(N = 4001) Safety		
2	Symptomatic VTE (nonfatal) and VTE-related death	41 (1.03)	55 (1.37)	−35 [−83 to 13]	−287
	Major bleeding	52 (1.30)	23 (0.57)	73 [30 to 115]	137
3	Nonfatal PE and VTE-related death	29 (0.73)	43 (1.07)	−35 [−76 to 6]	−287
	Critical site bleeding and fatal bleeding	19 (0.48)	11 (0.27)	20 [−7 to 47]	498
4	Nonfatal PE, CV/VTE-related death, MI, and nonhemorrhagic stroke	99 (2.48)	106 (2.65)	−17 [−87 to 52]	−580
	Critical site bleeding and fatal bleeding	19 (0.48)	11 (0.27)	20 [−7 to 47]	498
5	VTE-related death	19 (0.48)	30 (0.75)	−27 [−62 to 7]	−365
	Fatal bleeding^b^	11 (0.28)	5 (0.12)	15 [−5 to 35]	665
MAGELLAN Subpopulation					
		(N = 2419) mITT	(N = 2506) mITT		
1	Asymptomatic lower proximal DVT, symptomatic VTE (nonfatal), and VTE-related death	94 (3.89)	143 (5.71)	−182 (−301 to −63)	−55
	Major bleeding	13 (0.54)	9 (0.36)	18 (−20 to 55)	560
		(N = 3218) Safety	(N = 3229) Safety		
2	Symptomatic VTE (nonfatal) and VTE-related death	30 (0.93)	42 (1.30)	−37 [−88 to 14]	−272
	Major bleeding	28 (0.87)	21 (0.65)	22 [−20 to 64]	455
3	Nonfatal PE and VTE-related death	22 (0.68)	35 (1.08)	−40 [−86 to 6]	−250
	Critical site bleeding and fatal bleeding	12 (0.37)	9 (0.28)	9 [−18 to 37]	1061
4	Nonfatal PE, CV/VTE-related death, MI, and nonhemorrhagic stroke	81 (2.52)	88 (2.73)	−21 [−99 to 57]	−481
	Critical site bleeding and fatal bleeding	12 (0.37)	9 (0.28)	9 [−18 to 37]	1061
5	VTE-related death	15 (0.47)	26 (0.81)	−34 [−73 to 5]	−295
	Fatal bleeding^b^	7 (0.22)	4 (0.12)	9 [−11 to 30]	1067

Abbreviations: CI, confidence intervals; CV, cardiovascular; DVT, deep vein thrombosis; MI, myocardial infarction; mITT, modified intent to treat; NNH, number-to-treat to harm; NNT, number-to-treat to benefit; PE, pulmonary embolism; VTE, venous thromboembolism.

^a^ A negative number denotes the number of patients needed to be treated with rivaroxaban instead of enoxaparin to prevent one additional harmful event. A positive number denotes the number of patients needed to be treated with rivaroxaban instead of enoxaparin to observe one additional harmful event. NNT and NNH are calculated as the reciprocal of the corresponding risk differences, all decimals being rounded down for NNT and NNH. All events from randomization (reference day) to day 41 (inclusive) are included.

^b^ For one rivaroxaban patient bleeding was the adjudicated cause of death, although the patient’s reported bleeding events were adjudicated as major but not as contributing to death (fatal bleeding).

## Discussion

The principal finding of this study is that the use of 5 key risk factors for major bleeding may significantly improve the benefit risk profile of rivaroxaban for both in-hospital and extended thromboprophylaxis in medically ill patients using clinically meaningful endpoints. Importantly, major and fatal bleeding rates in the subpopulation compared to the original population in the MAGELLAN trial were significantly reduced, while anticoagulant efficacy was maintained. These findings have implications for both the safety and the benefit risk profile of extended thromboprophylaxis with DOACs across a broad group of hospitalized medically ill patients, of whom modeling estimates suggest that up to 25% to 30% of the medically ill population are at risk of post-discharge VTE.^21,25^

At day 35 when compared to the enoxaparin/placebo group, rivaroxaban produced a favorable benefit risk profile for all 5 pairs of clinically meaningful endpoints which included asymptomatic and symptomatic VTE and VTE-related death, major bleeding, and fatal and irreversible thrombotic and bleeding events, with NNTs ranging from 55 to 481 and NNHs ranging from 455 to 1067. If we assume that approximately 6 million patients in the US and the EU are at risk of postdischarge VTE events as modeling assumptions suggest,^21,25^ then a strategy of extended thromboprophylaxis to 35 days with rivaroxaban has the potential to prevent fatal or irreversible thrombotic events in approximately 12 000 patients annually and prevent symptomatic VTE and VTE-related death as well as nonfatal and fatal PE in approximately 24000 patients annually at the cost of one-half to one-fourth that number of major or fatal bleeding. These figures have important public health implications when looking at the morbidity, mortality, and health-care costs associated with hospital acquired VTE worldwide.^26^

There are clinical data supporting the increased risk of major bleeding with the 5 key risk factors as described in this analysis. Previous studies have reported that patients with bronchiectasis are at high risk of bleeding, as are patients who have had a history of recent bleeding and active gastroduodenal ulcer.^27,28^ Moreover, an elevated bleeding risk in patients on dual antiplatelet therapies has also been well described in the literature, including those with medical illness.^29^ Patients with active cancer are at increased bleeding risk as well as increased thrombotic risk and may benefit from a separate paradigm of primary thromboprophylaxis.^30,31^ The only bleeding risk assessment model—the IMPROVE model—which has been specifically validated in hospitalized medically ill patients incorporates many of the bleeding risk factors in the present study and corroborates our observation that approximately 10% to 20% of medically ill patients would be at high risk of bleeding from pharmacologic thromboprophylactic measures.^32,33^

An important observation comes from 2 recent randomized trials of acutely ill medical patients. The MARINER trial prospectively tested whether the 5 key additional exclusionary criteria found in this analysis could improve the benefit risk profile of rivaroxaban in a postdischarge setting.^17^ In this trial of over 12 000 patients that were randomized to rivaroxaban or placebo for 45 days postdischarge, the incidence of major bleeding was very low and not statistically significant between groups (0.3% vs 0.2%, Hazard Ratio = 1.88 (95% CI = 0.84, 4.23, *P* = .124). The incidence of critical site and fatal bleeding was 0.1% and <0.1% in the rivaroxaban group and <0.1% and 0.0% in the placebo group, respectively. Although MARINER did not meet its primary efficacy outcome, exploration of prespecified secondary outcomes suggested a significant reduction in symptomatic VTE alone and as a composite endpoint with all cause-mortality. A second study, APEX, with betrixaban in acutely ill medical patients also implemented very similar exclusion criteria and demonstrated a similar relative reduction in VTE and VTE-related death without an increase in major bleeding.^16^ Taken together, these studies suggest that an improved benefit risk profile can be obtained for extended thromboprophylaxis by treating patient at risk for VTE while carefully avoiding treating patients at high risk of bleeding.

This analysis has several strengths and limitations. First, this MAGELLAN subpopulation is a post hoc analysis. The 5 key factors associated with higher risk of bleeding were not part of the original study and this MAGELLAN subpopulation was retrospectively designed. However, the MARINER study, which prospectively used the same exclusionary criteria, established an acceptable safety profile of rivaroxaban in medically ill patients undergoing extended thromboprophylaxis. A direct comparison between the MAGELLAN subpopulation and MARINER is challenging, as the MARINER trial began treatment at posthospital discharge for 45 days, the primary efficacy endpoint was restricted to symptomatic VTE and VTE-related death, and there was a use of a dose adaption for patients with reduced creatinine clearance.^17^ Second, there is a possibility that we have not selected the most optimal window for each of these 5 factors. We excluded any bleeding and active gastroduodenal ulcer within 3 months of the randomization. The APEX study that investigated betrixaban for the same indication as MAGELLAN, used 5 similar key exclusion criteria albeit with a different window for some (eg, within 6 months of randomization for previous bleeding and within 2 years of randomization for gastroduodenal ulcer).^16^ We performed a sensitivity analysis of these 2 criteria in MAGELLAN using the APEX time window. Using bleeding within 6 months, no additional major bleeding events were identified, while using a 2-year window for the gastroduodenal ulcer criterion, only 1 additional major bleeding event was identified in a rivaroxaban patient. Third, it is possible that we have not accounted for hidden confounders in the subpopulation which may make our results less generalizable. However, this is unlikely given the fact that only 20% of patients with a balanced percentage of patients from the 2 treatment group arms were excluded from the original MAGELLAN population for the purposes of this analysis. Some historical data on bleeding prior to randomization may have been subject to recall bias. Fourth, we used the principal efficacy outcome of total VTE that included both asymptomatic proximal DVT (verified by screening ultrasonography), symptomatic VTE, and VTE-related death and major bleeding in our benefit risk calculations. Our previously explored rationale for the present study’s pairwise comparisons of efficacy and safety outcomes considered similar degrees of clinical severity and impact.^22^ Recent data from 2 trials of thromboprophylaxis in acutely ill medical patients have given support to the clinical significance of asymptomatic proximal DVTs identified during screening ultrasonography with a significant increase in mortality observed.^34,35^

In conclusion, 5 key risk factors for major bleeding in an acutely ill medical population that have an indication for thromboprophylaxis have been identified. Utilizing these key risk factors, we identified a subpopulation of MAGELLAN which excluded approximately 20% of the study population, and which substantially improved the benefit risk profile for in-hospital and extended thromboprophylaxis with rivaroxaban. The prospective use of these criteria in the MARINER study confirmed that they can help to identify a population in which rivaroxaban may be safely utilized for extended thromboprophylaxis. The present analysis can aid in the identification of medically ill patients with the greatest likelihood of benefit and the least risk of harm for extended thromboprophylaxis, which requires careful attention to both risk factors for VTE and for major bleeding.

## Supplemental Material

Supplemental Material, Spyropoulos.Supplementary_14_Aug_2019_final - Improved Benefit Risk Profile of Rivaroxaban in a Subpopulation of the MAGELLAN Study Click here for additional data file. Supplemental Material, Spyropoulos.Supplementary_14_Aug_2019_final for Improved Benefit Risk Profile of Rivaroxaban in a Subpopulation of the MAGELLAN Study by Alex C. Spyropoulos, Concetta Lipardi, Jianfeng Xu, Wentao Lu, Eunyoung Suh, Zhong Yuan, Bennett Levitan, Chiara Sugarmann, Yoriko De Sanctis, Theodore E. Spiro, Elliot S. Barnathan and Gary E. Raskob in Clinical and Applied Thrombosis/Hemostasis
