# Supplementary material for: Improved Benefit Risk Profile of Rivaroxaban in a Subpopulation of the MAGELLAN Study
Source: Clin Appl Thromb Hemost. 2019 Nov 20;25:1076029619886022. doi: 10.1177/1076029619886022 (PMC7019408; doi:10.1177/1076029619886022)
Supplement: Supplemental Material, Spyropoulos.Supplementary_14_Aug_2019_final - Improved Benefit Risk Profile of Rivaroxaban in a Subpopulation of the MAGELLAN Study [file Spyropoulos.Supplementary_14_Aug_2019_final.pdf]

Supplementary Table 1 Number of subjects per analysis set in MAGELLAN and MAGELLAN Subpopulation

Analysis set	MAGELLAN			MAGELLAN Subpopulation		
	Rivaroxaban	Enoxaparin	Total	Rivaroxaban	Enoxaparin	Total
Randomized	4050	4051	8101	---	---	---
Safety	3997	4001	7998	3218	3229	6447
mITT Day 35	2967	3057	6024	2419	2506	4925
PP Day 10	2938	2993	5931	2385	2433	4818
mITT Day 10	3232	3271	6503	2608	2659	5267
PP Day 35	2516	2586	5102	2066	2134	4200

mITT, modified Intent to Treat; PP, per protocol.

Supplementary Table 2 Reasons to exclude from the MAGELLAN Subpopulation (safety analysis set)

Exclusion criteria	Rivaroxaban	Enoxaparin/ Placebo	Total
	N = 779	N = 772	N = 1551
Active cancer at randomization	294 (37.7%)	290 (37.6%)	584 (37.7%)
Dual antiplatelet therapy at baseline	246 (31.6%)	233 (30.2%)	479 (30.9%)
Baseline bronchiectasis/ pulmonary cavitation	60 (7.7%)	61 (7.9%)	121 (7.8%)
Active gastroduodenal ulcer	116 (14.9%)	116 (15.0%)	232 (15.0%)
Bleeding in 3 months prior to randomization	126 (16.2%)	133 (17.2%)	259 (16.7%)

Patients who met high risk bleeding exclusion criteria.

Supplementary Table 3 Fatal Bleeding in MAGELLAN

Treatment Group	Duration of Study Drug	On-set of AE Study Day	Death Study Day	Age/Sex	Relevant Medical History	Bleeding Site
Rivaroxaban	2	3	3	62, Male	Bronchiectasis, COPD, septicemia, tuberculosis	Pulmonary
Rivaroxaban	8	8	9	52, Male	Bronchiectasis, COPD, hemoptysis, HF, kidney injury	Pulmonary
Rivaroxaban	11	12	13	44, Female	Active lung cancer, COPD	Pulmonary
Rivaroxaban	3	4	6	79, Male	Bronchiectasis, COPD, pneumonia	Gastrointestinal
Rivaroxaban[a]	5	5	8	70, Male	Afib, COPD, HF, HTN, MI, Renal insufficiency	Intracranial
Rivaroxaban[a]	33	33	34	85, Male	COPD, DM, HTN, pneumonia	Intracranial
Rivaroxaban[a]	11	11	22	70, Male	DM, HTN, liver steatosis	Retroperitoneal
Enoxaparin[a]	2	3	3	86, Male	Suspected bronchial neoplasm, HTN, HF, UTI	Tracheal

Afib, Atrial Fibrillation; COPD, chronic obstructive pulmonary disease; DM, Diabetes Mellitus; HTN, arterial hypertension; HF, Heart Failure; MI, myocardial infarction; UTI, Urinary Tract infection. [a]Fatal Bleeding in MAGELLAN subpopulation.
